# Developmental Principles: Fact or Fiction

**DOI:** 10.1100/2012/980151

**Published:** 2012-02-15

**Authors:** A. J. Durston

**Affiliations:** Sylvius Laboratory, Institute of Biology, University of Leiden, Wassenaarseweg 72, 2333 BE Leiden, The Netherlands

## Abstract

While still at school, most of us are deeply impressed by the underlying principles that so beautifully explain why the chemical elements are ordered as they are in the periodic table, and may wonder, with the theoretician Brian Goodwin, “*whether there might be equally powerful principles that account for the awe-inspiring diversity of body forms in the living realm*”. We have considered the arguments for developmental principles, conclude that they do exist and have specifically identified features that may generate principles associated with Hox patterning of the main body axis in bilaterian metazoa in general and in the vertebrates in particular. We wonder whether this exercise serves any purpose. The features we discuss were already known to us as parts of developmental mechanisms and defining developmental principles (how, and at which level?) adds no insight. We also see little profit in the proposal by Goodwin that there are principles outside the emerging genetic mechanisms that need to be taken into account. The emerging developmental genetic hierarchies already reveal a wealth of interesting phenomena, whatever we choose to call them.

## 1. The Basic Idea

While still at school, most of us are deeply impressed by the underlying principles that so beautifully explain why the chemical elements are ordered as they are in the periodic table and may wonder, with the theoretician Brian Goodwin [1], “whether there might be equally powerful principles that account for the awe-inspiring diversity of body forms in the living realm.” In fact, the question of how an organism acquires its structure and form during embryogenesis is one of the most intriguing and challenging questions in science. It is now becoming clear that there are indeed developmental principles. These principles define the developmental constraints that limit the life forms that can evolve. These constraints operate above and beyond the constraints imposed by Darwinian natural selection.

Many people have debated this point, for example, “Developmental constraints (defined as biases on the production of variant phenotypes or limitations on phenotypic variability caused by the structure, character, composition, or dynamics of the developmental system) undoubtedly play a significant role in evolution” [2], and “Darwin's theory of evolution by natural selection focuses on inheritance and survival without attempting to explain the forms organisms take. The first part of Form and Transformation looks critically at the conceptual structure of Darwinism and describes the limitations of the theory of evolution. A theory of biological form is needed to understand the structure of organisms and their transformations.” Forward to [3]. Not all agree: “we find ourselves perched on one tiny twig in the midst of a blossoming and flourishing tree of life and it is no accident, but the direct consequence of evolution by non-random selection” [4].

In fact, it is obvious that developmental principles or developmental constraints apply. The form of living organisms is generally predictable and limited. Animals cannot evolve to just anything. No metazoan, no matter how fast its movement, has yet evolved wheels. Pigs cannot fly.

## 2. Convergent Evolution

Another indicator about developmental constraints is the phenomenon of convergent evolution. Organisms in very different taxa can apparently independently evolve very similar structures, possibly suggesting that general principles apply. The best known example of this is the apparently independent evolution of the “camera eye” in vertebrates and cephalopod molluscs, while less complex members of the taxa concerned (nonvertebrate chordates and noncephalopod molluscs lack this complex structure and have simpler eyes). There are many other examples of convergent evolution, including a remarkable number of parallels between cephalopods and vertebrates [7]. There are three obvious possible explanations for convergent evolution.

1. The convergence is illusory. The gene set concerned is ancestral and evolved in a common ancestor but remained partly unused and cryptic until evolutionary manifestation of the phenotype. At first sight, this seems unlikely because it apparently requires evolution without natural selection—but the gene set concerned may have evolved for a different purpose. See below.
2. The same mechanism evolved independently in two different taxa. This is inherently unlikely and is not considered in the literature.
3. Two different mechanisms evolved in two different taxa but they deliver the same or a very similar phenotype. This is genuine convergent evolution.

**Figure 1:**
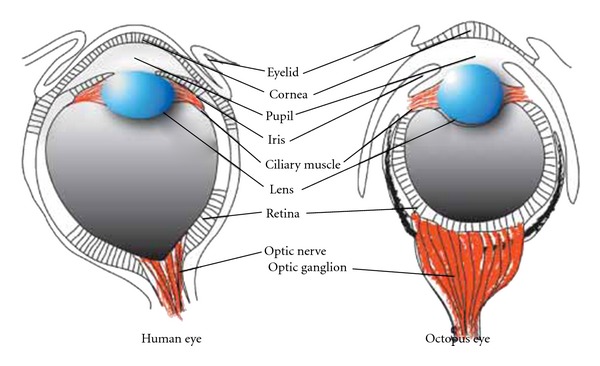
Human and octopus camera eyes have very similar morphology. Essentially the whole gene set needed to make this complex structure was already selected in the common ancestor of the bilaterians, long before camera eyes appeared. This poses a problem for “convergent evolution”. See Ogura et al. [14].

The necessary information to distinguish between these alternatives is not available for most examples of convergent evolution although alternative 3 is generally the null hypothesis. It is known, however, for eye development that the basic machinery for making an eye is universal for the metazoa [8–10]. This includes a cascade downstream of Pax6 and genes for photoresponsive proteins that were probably originally imported into metazoan cells from synergistic photosynthetic bacteria. It has generally been assumed that convergent evolution of the complex camera eye in vertebrates and cephalopods is due to alternative 3 (true convergent evolution). We now thus know that the basic mechanism for making an eye is ancestral and common to both taxa (alternative 1). The question arises whether the advanced special features of the camera eye evolved independently in vertebrates and cephalopods. Till recently, the consensus was yes [11–13]. However, a very interesting recent study by Ogura et al. [14] has challenged this view. These authors used bioinformatics to show that 729 out of 1052 (69.3%) of genes studied that were expressed in the octopus eye were also expressed in the human eye. In contrast, the expression similarity between human and octopus connective tissue was rather low. Ogura et al. also examined the availability of these eye genes in the sequenced genomes of a variety of other bilateria. The findings show that 1019 out of the 1052 genes (i.e., essentially all, presumably including the genes specifically needed for “camera” properties) had already existed (and were thus selected) in the common ancestor of bilateria, long before camera eyes appeared (Figure 1). This shows that there is a single ancestral gene set for the vertebrate and cephalopod camera eye. These findings possibly argue for alternative 1 and an ancestral mechanism as the driving force for this apparent example of convergent evolution. Obviously, the case is not complete. More needs to be done in studying different developmental stages in eye development and in understanding the developmental mechanisms involved, particularly those causing the known differences between vertebrate and cephalopod eyes. It is possible that while the relevant genes are conserved, the secret lies in their regulation and usage. We note that lens crystallins: typical “camera eye” genes, are a diverse group of proteins, some of which are either known to have other functions than in the eye lens or strongly resemble proteins that do [15].

In conclusion, genes expressed in the octopus eye belong to a gene set that was already selected in the ancestor of the first bilaterians. Some of these early selected genes were presumably concerned with specifying an ancestral eye. Others, that specify the advanced features of the octopus camera eye, were not selected for this function because camera eyes were not yet available in early bilateria. These must be dual-purpose genes that were originally selected for functions that were operational in primitive bilateria and were secondarily recruited later for camera eye development. The camera eye lens crystallins appear to have exactly such a dual nature, suggesting that this view is correct. The lens crystallins in vertebrates and cephalopods are also generally different, indicating secondary recruitment of different members of a larger gene set in these two versions of the camera eye. In contrast, some camera eye genes are identical in cephalopods and vertebrates [16]. These findings indicate a complex mechanism for this example of “convergent evolution”, with elements of explanations 1 and 3.

## 3. What Are Developmental Principles?

The nature of developmental principles has attracted discussion. “Do genes explain life? Can advances in evolutionary and molecular biology account for what we look like, how we behave, and why we die? In this powerful intervention into current biological thinking, Brian Goodwin argues that such genetic reductionism has important limits. Drawing on the sciences of complexity, the author shows how an understanding of the self-organizing patterns of networks is necessary for making sense of nature. Genes are important, but only as part of a process constrained by environment, physical laws, and the universal tendencies of complex adaptive systems. In a new preface for this edition, Goodwin reflects on the advances in both genetics and the sciences of complexity since the book's original publication” [17]. “While neo-Darwinism has considerable explanatory power, it is widely recognized as lacking a component dealing with individual development, or ontogeny. This lack is particularly conspicuous when attempting to explain the evolutionary origin of the thirty-five or so animal body plans, and of the developmental trajectories that generate them. This significant work examines both the origin of body plans in particular and the evolution of animal development in general. Wallace Arthur ranges widely in his treatment, covering topics as diverse as comparative developmental genetics, selection theory, and Vendian/Cambrian fossils. He places particular emphasis on gene duplication, changes in spatio-temporal gene-expression patterns, internal selection, coevolution of interacting genes, and coadaptation. The book will be of particular interest to students and researchers in evolutionary biology, genetics, paleontology, and developmental biology” [18]. In fact, although Goodwin and others are presumably right that dynamical stability, self-organisation, and physical laws are important and although this is a valid point of view, it is predictable that many developmental principles will be inherent in the developmental genetic machinery, that is, in the gene hierarchies that mediate development. Wallace Arthur is right. The theoretical approach to developmental principles can also, because of its lack of specific concrete detail, sometimes take on almost a religious tint, a fact that fails to inspire confidence and a point of view that evoked a strong response from the Darwinists. “The human eye is so complex and works so precisely that surely, one might believe, its current shape and function must be the product of design. How could such an intricate object have come about by chance? Tackling this subject—in writing that the New York Times called “a masterpiece”—Richard Dawkins builds a carefully reasoned and lovingly illustrated argument for evolutionary adaptation as the mechanism for life on earth”. See forward to [19].

## 4. An Example: The Hox Genes

An example of the evolution of a molecular mechanism and a possible source of some developmental principles is supplied by the well-studied Hox genes. What we see here is clusters of closely related genes for transcription factors that together mediate part of a developmental function. That function is patterning an embryonic axis and in all bilaterian metazoa, either one cluster of 10–13 Hox genes patterns part or all of the main body axis or 4 or 8 similar clusters act in parallel to do the same (in the vertebrates). A whole Hox cluster thus acts as a functional unit or metagene. No one Hox gene can pattern an embryonic axis but the genes in one cluster, acting together, can function in this [20, 21]. An important property that enables this is collinearity. The Hox genes in a cluster are expressed and act sequentially, from 3′ to 5′ to specify sequential levels in the body. This is called spatial collinearity and is evident in all bilateria. Hox clusters are thought to have evolved by tandem duplication of an ancestral ur-Hox gene and by sequential evolutionary modification of the duplicated genes. The individual Hox genes at the same homologous position in different clusters and different organisms thus have conserved properties. It is for example, possible to replace a *Drosophila*-Hox gene with a human Hox gene corresponding to the same cluster position. This functions correctly [22]. It is claimed in *Drosophila* that it is still possible to identify the present day Hox gene representing the evolutionary ground state-the ur-Hox gene [23].

Hox genes, their duplication, clustering, and spatial colinearity clearly provide universal developmental principles for axial patterning in the bilaterian metazoa. These seem to have evolved early. Among the bilaterian phyla, there are animals with clustered Hox genes and animals where the Hox cluster is in various stages of disintegration—from two pieces as in various *Drosophila* species [20] to scattered—“atomised” Hox genes, for example, in Oikopleura [24]. It is argued that clustering is ancestral and that scattered Hox genes have arisen by cluster disintegration. Interestingly, scattered Hox genes retain their spatial sequence of expression and action along the main body axis. The gene relationships that arose within the ancestral Hox cluster are thus preserved. No bilaterian metazoan has been detected with less than 10 Hox genes, so stages in the progress of Hox gene duplication and modification have not been preserved and this cannot be monitored.

There is a unique situation in the vertebrates. Here, Hox gene sizes, that are very large in invertebrates, have become small, so the Hox clusters are more compact. The basic Hox cluster size has also been amplified from 10 to 13 genes, by amplification of the number of posterior AbdB orthologues (10–13). The genome has also been duplicated twice or more during vertebrate evolution, so most tetrapod vertebrates have 4 similar Hox clusters, following 2 genome duplications, and teleost fishes and a few other vertebrates have 8 Hox clusters, due to 3 genome duplications [20]. The multiple vertebrate Hox clusters are essentially copies of each other and share functions but each of the 4–8 copies misses different specific Hox genes. This is most extreme in the zebrafish Hox Db cluster, where all of the Hox genes are missing and only the Hox associated micro-RNA gene Mir10 persists [25].

**Figure 2:**
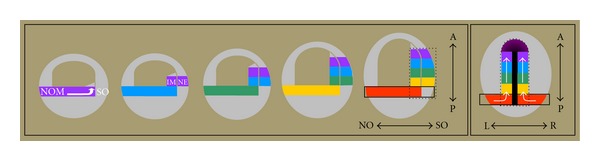
A developmental principle: time-space translation. The figure illustrates how temporally collinear Hox expression (in vertebrate gastrula mesoderm) is translated to a spatially collinear axial Hox pattern (in axial mesoderm and the neural plate). For a detailed explanation, see Durston et al [5, 6].

The unique vertebrate Hox clusters are associated with unusual Hox collinearity principles. Vertebrate Hox genes unusually show temporal collinearity. For example, in early development, Hox genes are first expressed in a time sequence during gastrulation [26]. This time sequence antedates and is used to generate the spatially collinear axial pattern of Hox expression in the early embryo, by a time- space translation mechanism [5, 6] (Figure 2). temporal collinearity and time-space translation represent vertebrate developmental principles associated with Hox patterning of the main body axis. These are what Maynard Smith et al. [2] define as local developmental constraints, while spatial collinearity and Hox gene duplication-clustering are universal constraints.

## 5. Conclusions

Above, we have considered the arguments for developmental principles and have specifically identified some features that could generate principles associated with Hox patterning of the main body axis in bilaterian metazoa in general and in the vertebrates in particular. We wonder whether this exercise serves any purpose. The features we have discussed were known to us as parts of developmental mechanisms and defining developmental principles (how, and at which level?) adds no insight. We also see little profit in the proposal by B. C. Goodwin that there are principles outside the emerging genetic mechanisms that need to be taken into account. The emerging developmental genetic hierarchies already reveal a wealth of interesting phenomena, whatever we choose to call them.
